# Multiple Bluish Nodules in a 20-Year-Old Female with Similar Lesions in Her Father

**DOI:** 10.1155/2019/7143876

**Published:** 2019-11-11

**Authors:** Punyawee Ongsri, Supenya Varothai

**Affiliations:** Department of Dermatology, Faculty of Medicine Siriraj Hospital, Mahidol University, Bangkok, Thailand

## Abstract

Glomuvenous malformations are congenital, benign, vascular malformations classified as subtypes of glomus tumors with predominant blood vessels, usually present at birth or childhood with multiple, bluish, soft papules and nodules or plaque-like cutaneous lesions. Later present with pronounced segmental lesions, superimposed on the primary lesions, suggesting type 2 segmental mosaicism. We present a rare case of familial glomuvenous malformations, a healthy young female presented with multiple bluish papules since birth which later developed dissemination later in her adolescence. Moreover, her father also had similar skin lesions on his left lower back.

## 1. Introduction

Glomuvenous malformations (GVMs) or previously termed glomangiomas are congenital, benign, vascular malformations, classified as a histological subtype of glomus tumors with predominant blood vessels [1, 2].

Glomus tumors are benign tumors or hamartomas of the perivascular structure, arise from modified smooth muscle cells, called glomus cells, located in the walls of the Sucquet-Hoyer canal, a specialized arteriovenous anastomosis central to thermoregulation [1, 2].

There are two forms of glomus tumor, solitary variant (90% of the cases) and multiple variant (10%) [3], while the solitary variant is usually painful and located in subungual area, the latter is mostly present in children with similar lesions in their family members, which is thought to be inherited in autosomal dominant fashion [1, 2].

Glomuvenous malformations usually present at birth as multiple, bluish, less than 1 cm in diameter, soft papules and nodules or plaque-like cutaneous lesions that are typically painful on compression. They slowly grow in size and number with age, involving in dermis and subcutis, with hyperkeratotic and cobblestone appearance, affecting predominantly extremities.

Histologically, GVMs appear as distended vein-like malformed vascular channels surrounded by one or more layers of glomus cells; uniform blue cuboidal cells with plump nuclei and eosinophilic cytoplasm [4]. These glomus cells demonstrated smooth muscle characteristics which are stained positively for vimentin and *α*-smooth muscle actin and negatively for desmin [5].

## 2. Case Presentation

**Figure 1:**
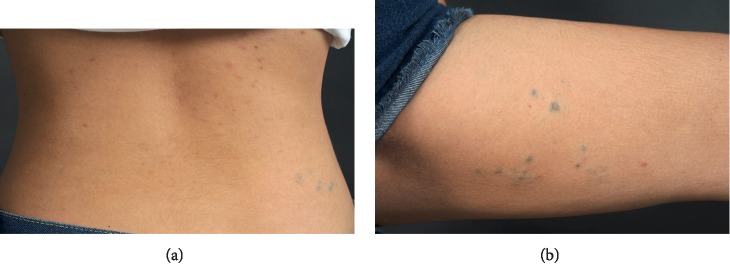
Clinical presentation of a 20-year-old female with multiple discrete soft, non-compressible painful bluish papules and nodules on her right lower back (a) and left inner thigh (b).

**Figure 2:**
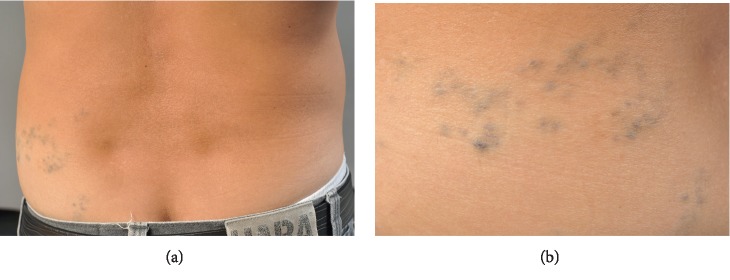
Her father also had similar skin lesions on his left lower back (a) and flank (b).

A 20-year-old Thai female from Chonburi, presented with multiple bluish papules on left thigh since birth. She noticed some new lesions later developed on her right lower back ten years ago. The lesions were asymptomatic until the last 2 months, they became painful on palpation (Figure 1). She was otherwise healthy. Her father also had similar skin lesions on his left lower back (Figure 2). He did not recognize the exact onset but they became more apparent in his childhood. Unlike his daughter, his lesions were asymptomatic and did not grow bigger in size or numbers.

Physical examination revealed multiple discrete soft, noncompressible painful bluish papules and nodules on her right lower back and left inner thigh. She denied episodes of bleeding from the cutaneous lesions or gastrointestinal tract.

**Figure 3:**
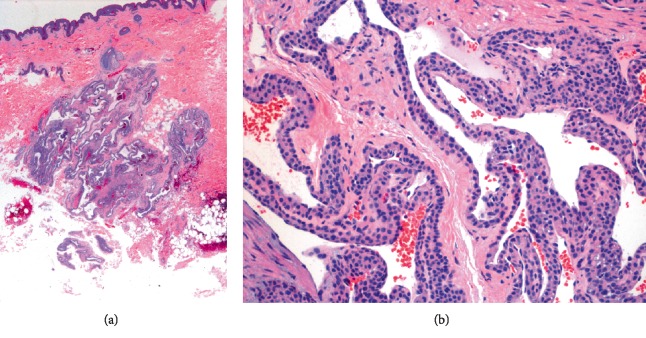
Histological study (hematoxylin and eosin staining) demonstrated large dilated, blood-filled vascular channels in the dermis (a), surrounded by layers of glomus cells (b).

Computed tomography angiography (CTA) of lower abdomen and left thigh revealed multiple discrete small cutaneous and subcutaneous enhancing nodules at the anteromedial aspect of left upper thigh, size up to 1 cm, and no evidence of vascular malformations. The excisional skin biopsy was done on her left thigh and sent for pathology. Histological examination revealed large dilated, blood-filled vascular channels, surrounded by layers of glomus cells in the dermis (Figure 3).

Laboratory tests revealed normal complete blood count and coagulation. Her blood chemistry tests are otherwise within normal limits.

After the diagnosis was made, the prognosis and treatment options were informed to the patient. She does not have a cosmetic concern and seeks no further treatment.

## 3. Discussion

Glomuvenous malformations (GVMs) mostly present since infancy or childhood without gender predilection [4], multiple lesions usually develop and disseminate later in lives in different sites from the initial lesions. They are often asymptomatic, but some lesions may be tender upon compression. Unlike, glomus tumors which are usually solitary benign vascular lesions predilected for a subungual area with predominance on the ring finger, present in adulthood and almost always painful and sensitive to changes in temperature [6].

GVMs are caused by loss of function mutations in the glomulin gene (GLMN, at chromosome 1p21p22) which encodes glomulin protein [7]; a component of a multiprotein complex utilized in vascular morphogenesis. Despite somatic mutations in sporadic cases, familial GVMs were reported with an autosomal dominant transmission with incomplete penetrance and variable expressivity [5]; penetrance varies from 80% at 20 to about 100% at age 30 years [8]. In addition, somatic second-hit mutations can alter the size and number of the lesions [9].

Some patients with familial or congenital multiple GVMs may later present with pronounced segmental lesions, arranged in a flag-like pattern, superimposed on the ordinary, non-segmental phenotype, suggesting type 2 segmental mosaicism (T2SM). Like our case, we present an otherwise healthy young female presented with GMV lesions since birth which later developed dissemination later in her adolescence. There have been less than 15 cases of congenital multiple GVM with type 2 segmental manifestation reported to date [10, 11].

In the past, GVM was considered a subtype of venous malformations (VMs) but recently proposed as a distinct entity according to its different clinical characteristic, histopathological findings, prognosis and treatment responses. Other clinical differential diagnosis includes blue rubber bleb nevus syndrome, Mafucci syndrome and congenital plaque-like blue nevi [3, 5]. While eccrine spiradenoma, leiomyoma, and angiolipoma are also in differential diagnosis but they usually occur later in life [12].

Venous malformations (VM) are compressible, bluish lesions that affect deeper tissue. They shrink when externally compressed or subjected to an antigravitational posture. Phleboliths and stasis can be associated with pain. Unlike GVMs, which are less compressible and painful under compression. D-dimer elevation can also be found more commonly in VM [13]. Magnetic resonance imaging (MRI) was the best investigation to determine the extent of the GVM lesions and their relationship to other anatomic structures [14]. Doppler ultrasound may demonstrate the slow-flow nature of GVMs, and the higher cellularity and weaker compressibility compared with VM [15]. However, histological examination is crucial to distinguish GVMs from ordinary VMs, despite distinctive clinical features [4].

Blue rubber bleb nevus syndrome (BRBNS) is a rare congenital disorder associated with double (cis) somatic activating TEK mutations [4, 16, 17], characterized by multifocal cutaneous and visceral venous malformations. Multiple easily compressible cutaneous rubbery blue nodules since birth are the characteristic presentation, whereas gastrointestinal bleeding resulting in iron deficiency anemia is an extracutaneous complication associated with significant morbidity and mortality [5].

Mafucci syndrome is an extremely rare, sporadic, and nonhereditary disease characterized by multiple enchondromas and subcutaneous hemangio-endotheliomas localized on extremities especially on fingers and toes [5]. These lesions can cause fractures, deformities, pain, and undergo malignant transformation. Association with heterozygous mutations in *IDH1* (NADP(+)-dependent isocitrate dehydrogenase 1 gene) has been identified, with the same mutation found in enchondromas and spindle cell hemangiomas [4, 16, 18].

The goals of treatment in GVMs are mainly aesthetic considerations, pain relief, functional improvement and prevention of complications. Current treatment options include surgical excision, sclerotherapy, and laser.

Unlike VM, GVMs, especially plaque-type, responded poorly to sclerotherapy or embolization. Surgical excision is less appropriate due to their extensive and multifocal nature, additionally, it could result in disfiguring scars and demonstrated 10% of recurrence rate [19].

Many case reports and studies demonstrated satisfying efficacy using laser treatments on GVM lesions. Dual-wavelength Pulsed Dye Laser and Neodynium-doped Yttrium Aluminium Garnet laser (PDL-Nd:YAG) showed the most promising results in 60% of size reduction and improvement in color with safety profile [20], the youngest patient successfully treated was reported of a 6-month-old girl, with less cutaneous adverse effects compared to Nd:YAG alone, the latter more frequently resulted in ulceration and scarring, especially in children [21].

The mechanism of its effectiveness is that the laser system provides deeper penetration of the tissue to the lesions of GVMs, of which affect both the dermis and hypodermis. PDL allows greater absorption of the Nd:YAG laser due to the production of methemoglobin via hemoglobin, requiring less fluencies of the latter and therefore a decreased risk of adverse effects [19]. Focal GVM lesions showed better clinical outcome than plaque-like lesions treating with this laser system, while the latter had a greater risk of ulceration and atrophic scarring [20].
